# Antimicrobial and anti-inflammatory activity of *Terminalia arjuna*

**DOI:** 10.6026/97320630019184

**Published:** 2023-02-28

**Authors:** Vijayalakshmi R, Ambalavanan N, Rajeshkumar S, Jaideep Mahendra

**Affiliations:** 1 Department of Periodontology, Meenakshi Ammal Dental College and Hospital, Maduravoyal, Chennai, India; 2 Nanobiomedicine Lab, Saveetha dental college and Hospital, SIMATS, Saveetha University, Chennai, India

**Keywords:** *Terminalia arjuna*, local drug delivery, chronic periodontitis, antimicrobial, anti-inflammatory

## Abstract

*Terminalia arjuna* is one of the important herbal plants with cardioprotective, antihyperlipidemic activity and many more applications. In the present investigation, In order to find out the protective role, we prepared aqueous and ethanolic extract of *Terminalia arjuna*. The objective of this study was to comparatively evaluate the antimicrobial and anti-inflammatory activity of aqueous and ethanolic extract of *Terminalia arjuna* and to compare between the two. Antimicrobial activity of the plant extract was assessed by using agar well diffusion method against oral pathogens. The anti-inflammatory activity of prepared *Terminalia arjuna* plant extract was analyzed using egg albumin denaturation assay. The results of this study, showed that both aqueous and ethanolic extract had very good antimicrobial activity against *Staphylococcus aureus*, *Pseudomonas species* followed by *Escherichia coli.* However, the aqueous extract showed higher anti-inflammatory activity when compared with ethanolic extract. So it can be concluded that aqueous extract of *Terminalia arjuna* could be used as a local drug delivery agent in the treatment of chronic periodontitis.

## Background:

Periodontal disease is a chronic inflammatory disease of the supporting tissues of the teeth, characterised by resorption of the alveolar bone and loss of soft tissue attachment. It is widely known that the cause of periodontal disease is a localised bacterial infection accompanied by a pathogenic microflora inside the periodontal pocket [1]. Mechanical debridement such as scaling and root planing has been a part of conventional treatments for periodontal disease in order to remove the subgingival flora and create root surfaces that are clear, smooth, biologically compatible, and is sufficient to stop periodontal destruction in most of the cases [2]. Given the bacterial etiology and the inflammatory pathogenesis of periodontitis, the adjunctive use of systemic antimicrobials and/or host response‐modulating medications has been proposed.But the systemic administration of drug causes many side effects like hypersensitivity, gastrointestinal intolerance, and the development of bacterial resistance. Also, another disadvantage is that they could not achieve adequate concentration at the site of action for a sufficient period. These problems would be significantly diminished if localised antibacterial drugs could be employed as they achieve 100 times higher concentrations in the sub gingival area [3]. In 1979, Max Goodson developed the concept of controlled release-local drug delivery [4], to overcome the challenges associated with systemic therapy and to limit the drug to its target site. (Finkelman RD, 1998) [5]. The use of natural products has served as a major source of drugs for centuries and is well established in some cultures, especially in Asia, America, and Africa. The advantages of herbal therapy are that it has preventive effects, stimulates the regulatory action of the defensive functions of the body and prepares the body for possible activity against external agents [6,7]. *Terminalia arjuna*, also known as *Arjuna*, *Dhavala*, *Kaubha*, *Nadisaraja*, *Veeravrikskha*, *Partha*, and *Indradru* is a medicinal plant indigeneous to India and belongs to the family of Combretaceae. The word "*Arjuna*" is used in the Rigveda to denote pure fame and silvery brightness, or white colour. It may be the first reference of *Arjuna* used as medicine stated in chief or principal sutra volume of Atharvaveda, Kaushiksutra (Sharma P C et al 2000) [8]. *T. arjuna* is a reasonably large, evergreen, deciduous tree that can reach heights of 20 to 25 metres and can be found all over India. The bark of *T. arjuna* is soft and thick, with an outside that is grey in colour and an inner side that readily flakes off in flat, substantial chunks. Bark of *T.arjuna* has been used widely in traditional system of medicine for various purposes such as to decrease cholesterol, reduce hypertension, reduce blot clots, and prevent coagulation of blood. Triterpenoids and other phytoconstituents, like tannins and flavonoids, have been shown to be useful in treating cardiovascular disorders. Active substances such ethyl gallate, gallic acid, arjunolic acid, ellagic acid, and flavones are extracted from the plant's leaves, bark, and fruits and tested for their potential to fight periodontal bacteria [9]. Therefore, it is of interest to evaluate the antimicrobial and anti-inflammatory activity of aqueous and ethanolic extracts of *T. arjuna*.

## Materials and Methods:

The study was approved by Institutional Ethical Committee of Meenakshi Ammal Dental College and Hospital (MADC/IEC-I/03/2022). The antimicrobial activity of the plant extract was tested by using agar well diffusion method against oral pathogens. The anti-inflammatory activity of prepared *T. arjuna* plant extract was analyzed using egg albumin denaturation assay.

## Preparation of plant extracts (Figure 1a):

Bark of disease-free plants was carefully selected for the study. It was cut into small pieces, shade dried for 10-15 days. Upon air drying at room temperature, it was grounded into fine powder under sanitized conditions.

## Preparation of aqueous extract (Figure 1b and c):

About 2gm air-dried powder of *T. arjuna* was mixed with 100 ml sterile distilled water and boiled for 15-20 minutes at 70° C. It was filtered with tea filter and allowed to cool. This extract was filtered using Whatman No. 1 filter paper and boiled again to get concentrated extract. It was further reduced to get concentrated 5mL aqueous extract. It was refrigerated at 4°C for future use.

## Preparation of ethanolic extract (Figure 1b and c):

About 2gm air-dried powder of *T.arjuna* powder was mixed with 100 mL sterile distilled water and boiled for 15-20 minutes at 70° C. It was filtered with tea filter and allowed to cool. This extract was filtered using Whatman No. 1 filter paper and boiled again to get concentrated extract. It was further reduced to get concentrated 5mL ethanolic extract. It was refrigerated at 4°C for future use.

## Antimicrobial activity:

**Figure 2:**
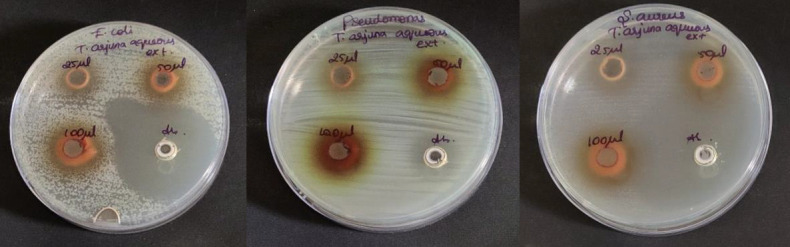
Antimicrobial activity of *T.arjuna* aqueous extract

**Figure 3:**
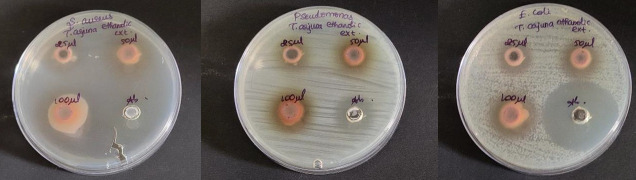
Antimicrobial activity of *T.arjuna* ethanolic extract

The antimicrobial activity of *T.arjuna* aqueous and ethanolic extracts at concentrations 25µl, 50µl and 100µl were tested against oral pathogens such as *S. aureus*, *E. coli* and *Pseudomonas sp* by agar well diffusion method (Figure 2a,b,c ,Figure 3a,b,c) [10,11]. Mueller Hinton agar was prepared to determine the zone of inhibition and sterilized for 45 minutes at 120 lbs. Media was poured into the sterilized plates and allowed to stabilize for solidification. A sterile 9mm polystyrene tip was used to cut the wells. The T,arjuna aqueous and ethanolic extracts with various concentrations were loaded into wells. The test organisms were swabbed. The plates were incubated at 37°C for 24 hours. Amoxicillin served as the control antibiotic. After the incubation time, the zone of inhibition was measured. The experiment was done in triplicate to avoid manual error.

## Anti-inflammatory activity:

1 ml of the *T.arjuna* aqueous and ethanolic extract was added to 10-20 ml distilled water at various fixations (10µL,20µL,30µL,40µL,50µL) and this was added to 0.45 mL egg albumin (1% aqueous solution ) and the pH of the mixture was acclimated to 6.3 with 1N hydrochloric acid. The specimens were then incubated at room temperature for 20 minutes. The samples were then heated at 55°C in a water bath for 30 minutes. The samples were cooled following which the absorbance was measured spectrophotometrically at 660 nm. Diclofenac sodium was used as the standard and Dimethyl Sulfoxide [DMSO] was used as the control. Protein denaturation was determined with the help of the following equation:

%inhibition = Absorbance of control- Absorbance of sample/ Absorbance of control x 100

##  Results:

**Figure 4:**
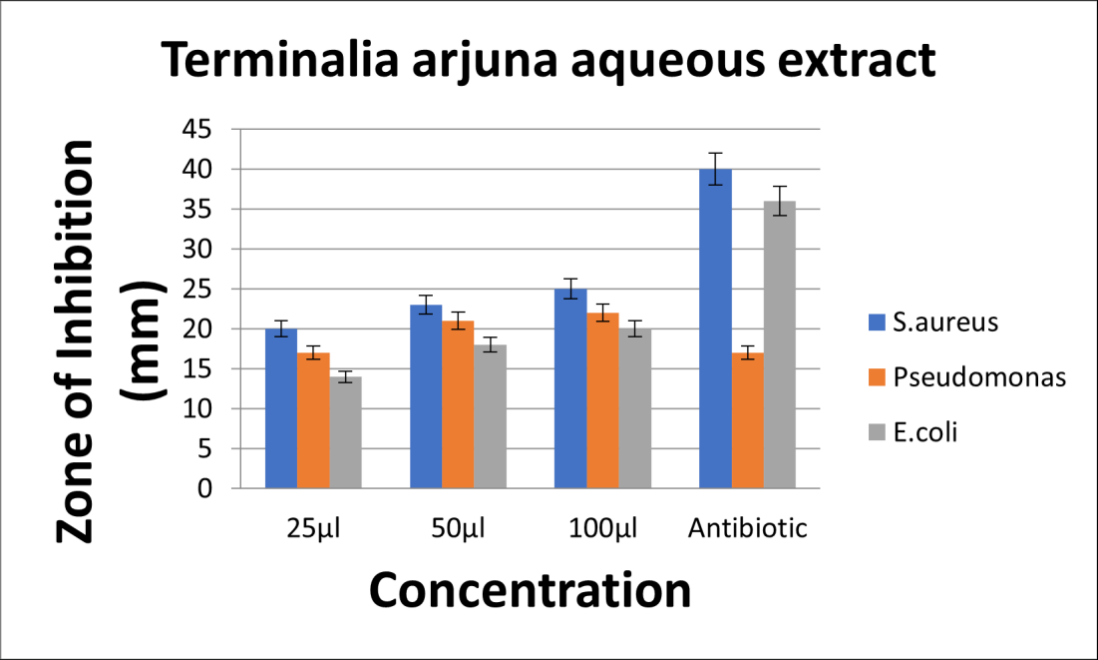
Graph depicting antimicrobial activity of aqueous extract of *T.arjuna*

**Figure 5:**
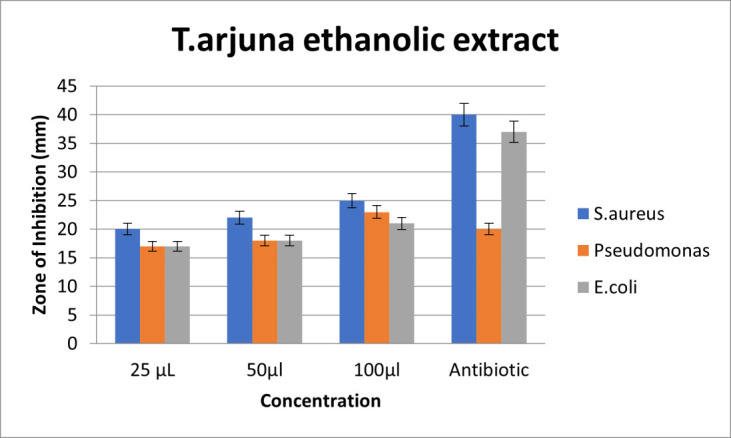
Graph depicting antimicrobial activity of ethanolic extract of *T.arjuna*

The aqueous extract of *T.arjuna* showed high zone of inhibition for S.aureus, followed by *Pseudomonas species* and E.coli at all concentrations. So, it can be interpreted that S.aureus has the highest sensitivity and E.coli has resistance against *T.arjuna* aqueous extract. Also, on comparison with standard(amoxycillin), aqueous extract of *T.arjuna* has effective antimicrobial activity at much lower concentration. Also, the antimicrobial activity of aqueous extract of *T.arjuna* exhibits antimicrobial activity in a dose dependent manner (Figure 4). Upon comparison, the ethanolic extract of *T.arjuna* showed high zone of inhibition for S.aureus, followed by similar zones of inhibition for *Pseudomonas species* and E.coli at 25µl and 50µl concentrations. At 100µl concentration, the ethanolic extract of *T.arjuna* showed high zone of inhibition for S.aureus, followed by *Pseudomonas species* and E.coli. So, it can be interpreted that S.aureus has the highest sensitivity; *Pseudomonas species* and E.coli have resistance against *T.arjuna* ethanolic extract at 25 and 50µl concentration. At 100 µl concentration, S.aureus has the highest sensitivity and E.coli has resistance against *T.arjuna* ethanolic extract. Also, on comparison with standard(amoxycillin), ethanolic extract of *T.arjuna* has effective antimicrobial activity at much lower concentration. Also, the antimicrobial activity of ethanolic extract of *T.arjuna* exhibits antimicrobial activity in a dose dependent manner (Figure 5).

## Comparison of antimicrobial activity of aqueous and ethanolic extracts of *T. arjuna* (Figure 2b and 3b)

**Figure 6:**
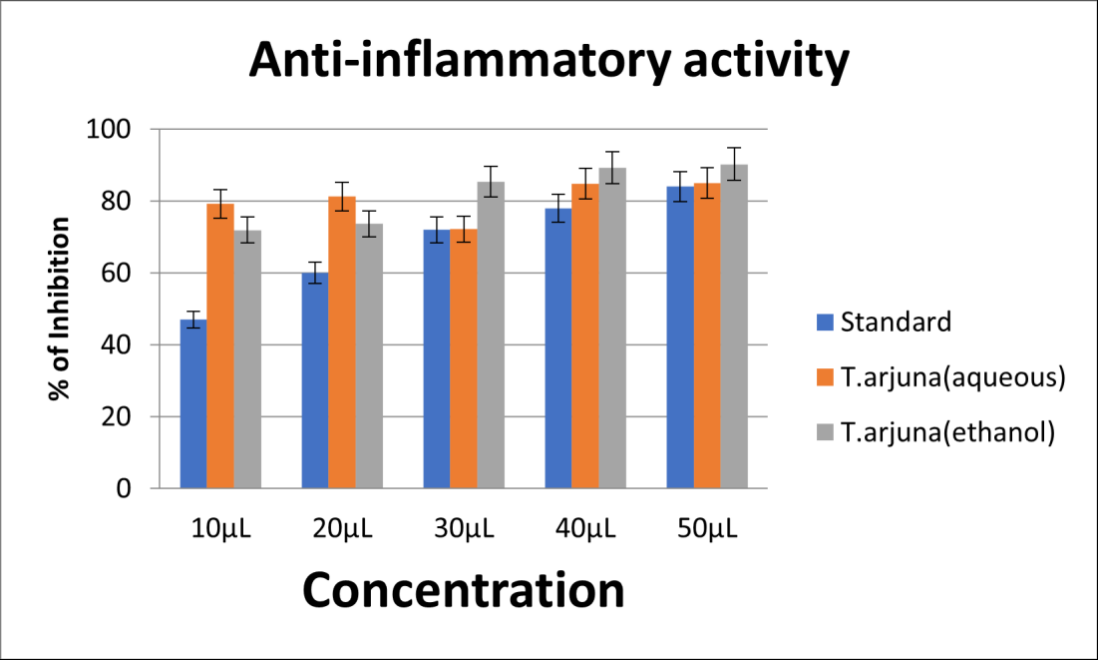
Anti-inflammatory activity of aqueous and ethanolic extracts of *T.arjuna*

Upon comparison between the aqueous and ethanolic extracts, both the aqueous and ethanolic extracts showed very good antimicrobial activity for S.aureus, *Pseudomonas species* followed by E.coli at dose dependent manner. Both extracts exhibited potent antimicrobial activity on comparison with control. S.aureus showed highest sensitivity to both aqueous and ethanolic extracts. Psuedomonas sp and *E. coli* showed relative resistance to both aqueous and ethanolic extracts. The anti-inflammatory activity was assessed by egg albumin denaturation assay at 10,20,30,40 and 50µL concentrations. Diclofenac sodium was taken as the control. At 10 and 20 µl concentrations, aqueous extract showed highest anti-inflammatory action. At 30,40 and 50µl concentrations, ethanolic extract showed highest anti-inflammatory action. Both the aqueous and ethanolic extracts showed potent anti-inflammatory action compared to standard. The aqueous extract showed higher anti-inflammatory activity at low concentrations, when compared with ethanolic extract (Figure 6).

## Discussion:

Antimicrobial screening of traditional medicinal plants has been the source of innumerable therapeutic agents. The factor important for antimicrobial treatment includes sensitivity of the infecting microorganism to a particular agent and it should target biochemical features of the invading pathogens that are not possessed by the normal host cell [12]. Arthur HR screened plant extracts and found that 20% of the species yielded positive reactions for alkaloids, 25% species contained steroids/triterpenoids and 45% of species possessed saponins [13]. The useful major groups of antimicrobial phytochemicals can be divided into alkaloids, flavones (flavonoids, flavonols, Quinones), essential oils, lectins, polypeptides, phenolics, polyphenols, tannins and terpenoids. The antibacterial activity could also be due to various chemical components and the presence of essential oils in adequate concentrations, which damage microorganisms [14]. The useful phytoconstituents of *T.arjuna* are: Triterpenoids, β-sitosterol, flavonoids, and glycosides. Luteolin in *T. arjuna* is responsible for antimicrobial action and it is effective against both gram positive and negative organisms [15]. Our study is in accordance with the study by Perumalsamy et al. [16] who showed that aqueous extracts of *T.arjuna* holds significant antibacterial activity against *E. coli*, Klebsiella aerogenes, Proteus vulgaris and Pseudomonas aeruginosa. Also, our study is in accordance with the study by Kamal Rai Aneja [17], where the aqueous extract of *T.arjuna* bark exhibited good activity against S,aureus. However, in our study, antimicrobial activity was concentration dependent, which was in accordance with Ramya et al. [18]. Our study is in accordance to the study by Sadika Akhter et al. [9], where ethanolic extracts showed potent antimicrobial activity at higher concentrations. Also, it is in accordance with Smith RA [19], who screened 50% ethanol extracts of 285 plant materials and revealed effective antibacterial properties. Also, the results were consistent with earlier studies of Alam et al, Phatak et al and Siva Sai P Dandu et al. [20,21, 22] which detailed broad range antibacterial activity possessed by bark and organic extracts of *T.arjuna*. However, in our study the ethanolic extract had limited antimicrobial activity compared to the aqueous extract, which was in accordance with the study by Kamal Rai Aneja. The limited spectrum of antimicrobial activity in the ethanolic extracts may be due to three reasons: (Kamal Rai Aneja 2012): firstly the polarity of antibacterial compounds make them more readily extracted by organic solvents as compared to aqueous extract; Secondly active compound may be present in insufficient amount in the crude extract to show activity with the dose level employed; thirdly if the active principle is present in high quantities, there could be other constituents present in the extract exerting antagonistic effects of the bioactive compounds [17]. Also, a similar study by Kumar et al. [23] found that the *T. arjuna* bark and leaves ethanolic extract and its different solvent fraction to show strong antimicrobial activity against Bacillus subtilis, *Staphylococcus aureus*, Eschericia coli, Klebsiella pneumonia, Pseudomonas aeruginosa and Salmonella typhi, respectively. Our study is in accordance with Jethinlalkhosh et al [24,^25^] who assessed the antibacterial activity of *T. arjuna* bark aqueous and methanolic extract by using the agar gel diffusion method against Escherichia coli, Klebsiella sp., *Pseudomonas sp*. and Staphylococcus sp. Aqueous and methanolic extract of *T.arjuna* showed inhibition against all the mentioned organisms in a dose dependent manner. Similarly, strong antibacterial activity was shown by the methanol extracts of *T. arjuna* against multi drug resistant Salmonella typhi [26]. The flavonoids in *T.arjuna* are responsible for the anti-inflammatory action, which bring about a reduction in inflammatory cytokines-hs CRP, IL-6,18, TNF-alpha [27,28]. Anti-inflammatory activity of *T. arjuna* bark powder was investigated and proved by Halder et al. [29]. Sharma et al found *T.arjuna* to have anti-inflammatory effect; the proposed mechanism was by inhibiting the enzyme cyclooxygenase (COX) leading to inhibition of prostaglandin synthesis [30]. Alam Morshed [20] assessed the anti-inflammatory, analgesic and cytotoxic properties of 50% ethanol extract of the stem bark of *T.arjuna* plant on laboratory animal; the stem bark of *T.arjuna* exhibited anti-inflammatory and analgesic activities. The mechanisms of anti-inflammatory activity may be related to the antiphlogistic action of the tannins. Flavonoids and other phenolics compounds of plant origin have been reported as antioxidants and as scavengers of free radicals. Antioxidants can also exert anti-inflammatory effects. Our results are in accordance with study by Biswas et al. [31], where he used methanol extract obtained from leaves of *T.arjuna* and found out good anti-inflammatory activity in Westar Albino rat model, which was found to be the most effective at higher concentrations employed. He stated that a more extensive study is necessary to determine the exact mechanism of action of the extracts and its active compound.

## Conclusion:

In this study, both aqueous and ethanolic extract showed very good antimicrobial activity; but the aqueous extract showed very good antimicrobial activity and higher anti-inflammatory activity even at low concentations when compared with the ethanolic extract. More detailed investigation at molecular, cellular levels with suitable animal models and human clinical studies are necessary to further elucidate other biological activities of *T.arjuna*.

## Figures and Tables

**Figure 1:**
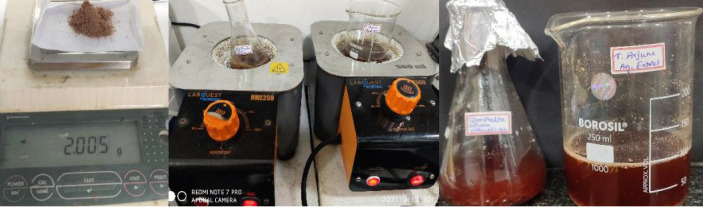
Preparation of aqueous and ethanolic extract of *T.arjuna*
